# Supplementary material for: The Role of ENHO in Pancreatic Adenocarcinoma: A Bioinformatics Approach
Source: Cancers (Basel). 2025 Jun 25;17(13):2139. doi: 10.3390/cancers17132139 (PMC12248484; doi:10.3390/cancers17132139)
Supplement: Supplementary file 1 [file cancers-17-02139-s001.zip › Figures S1-S4.pdf]

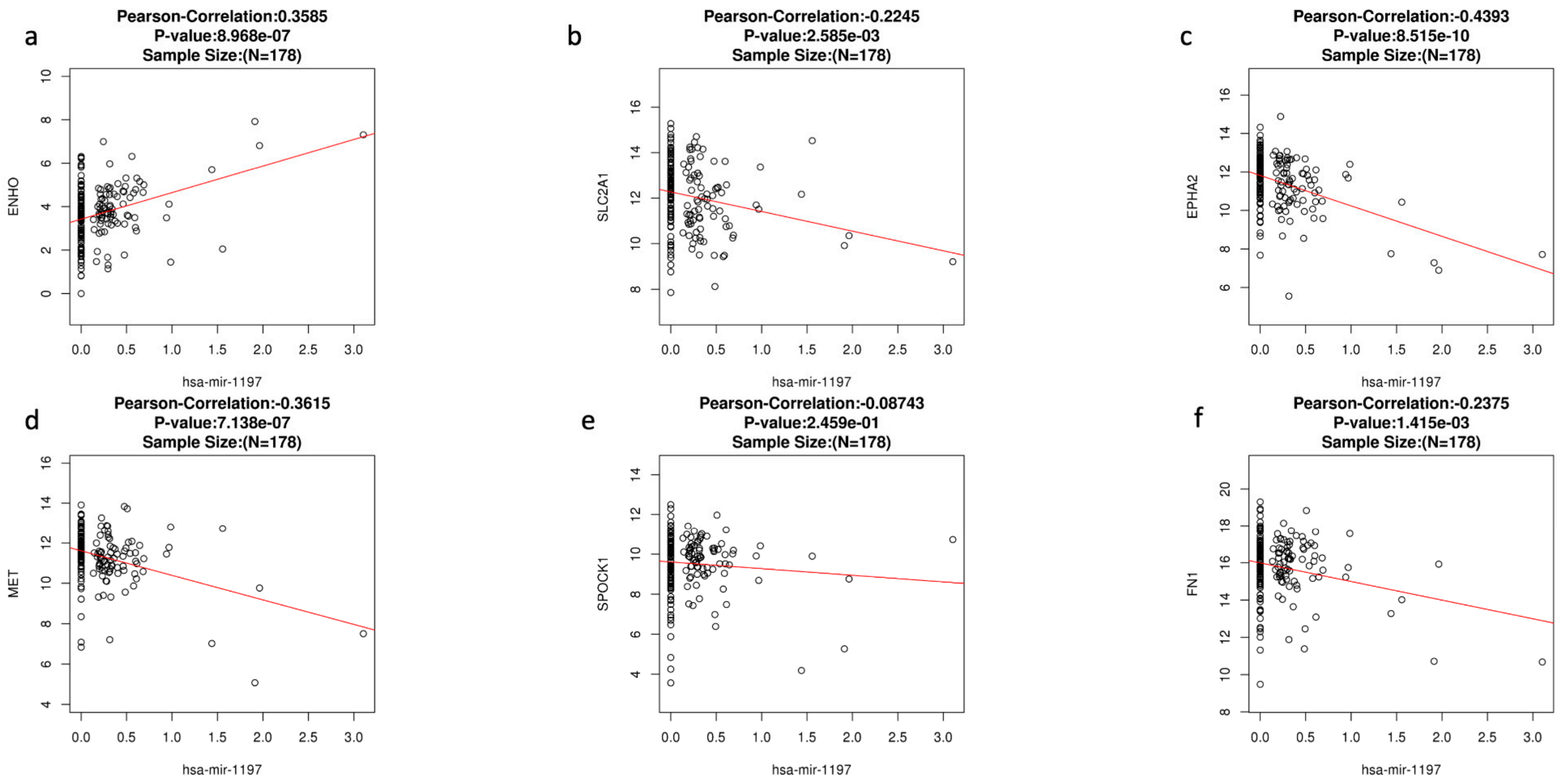

Figure S1 showing the correlation of mir-1197 with its network interaction in Figure 7. a) Shows the correlation between mir-1197 and *ENHO*. b) Shows the correlation between mir-1197 and SLC2A1. c) Shows the correlation between mir-1197 and EPHA2. d) Shows the correlation between mir-1197 and MET. e) Shows the correlation between mir-1197 and SPOCK1. f) Shows the correlation between mir-1197 and FN1.

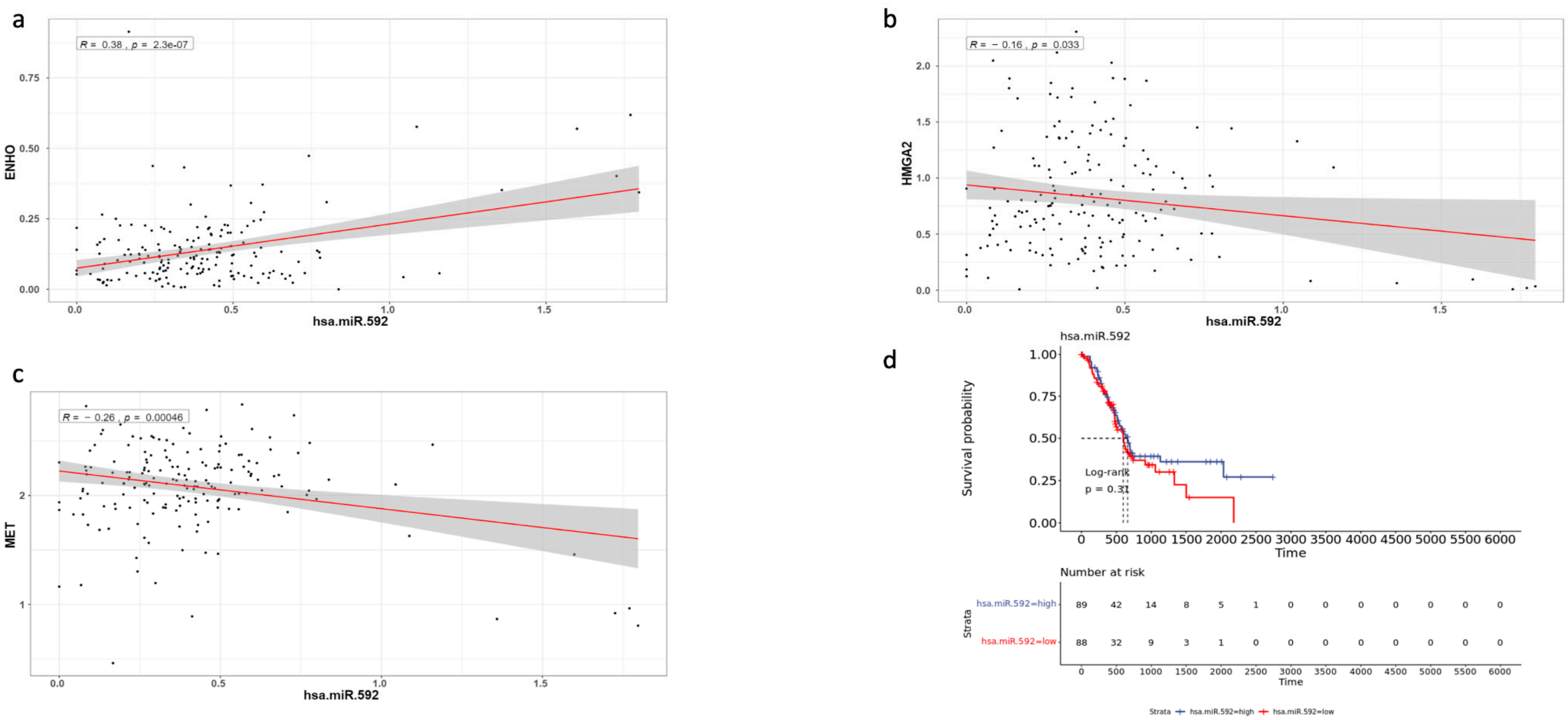

Figure S2 validates the association between *ENHO* and MiR-592. a) Shows the correlation between *ENHO* and MiR-592. b) Shows the correlation between HMGA2 and MiR-592. c) Shows the correlation between *MET* and MiR-592. d) Kaplan Meier plot of OS for MiR-592.

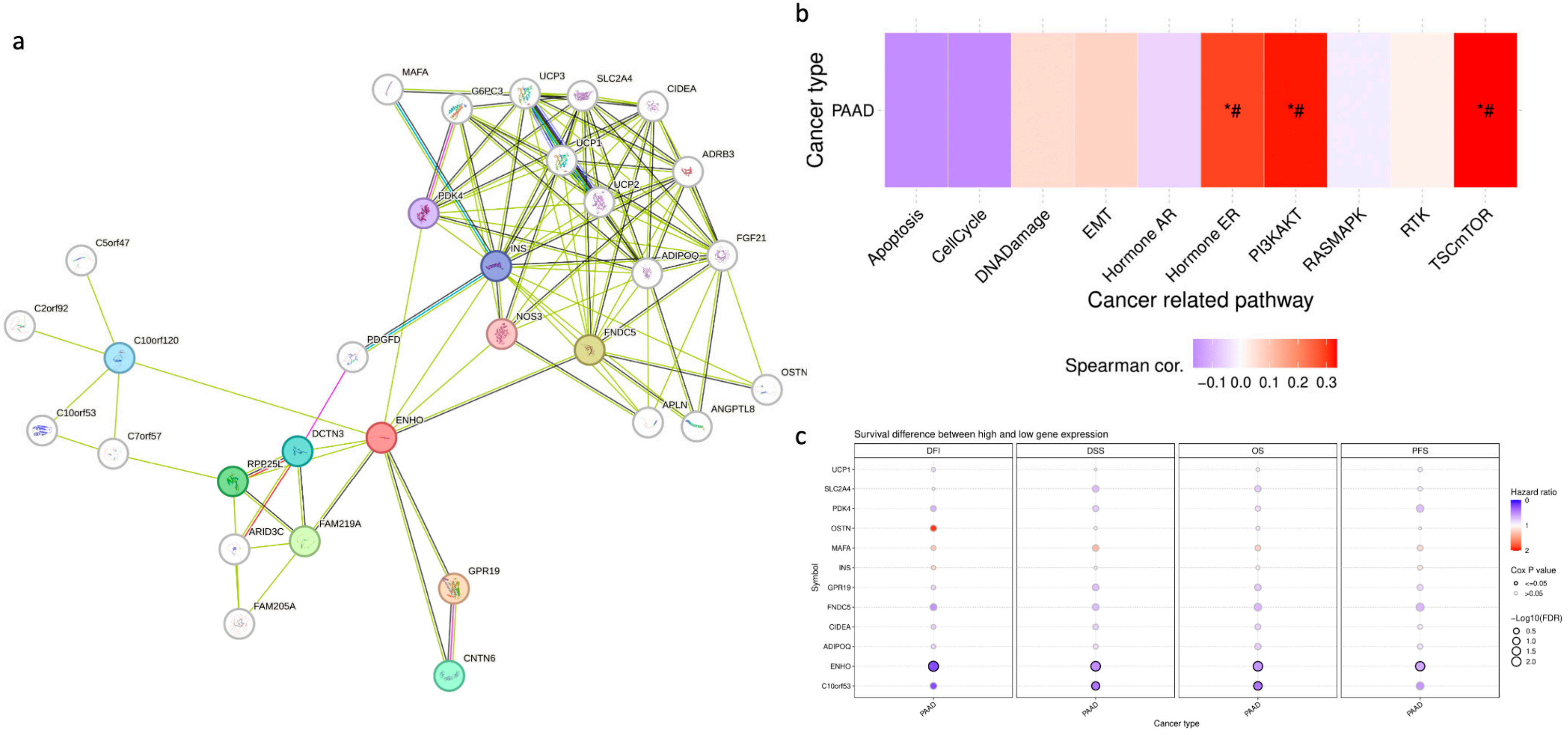

Figure S3 shows the Protein-to-protein interaction network for *ENHO*. a) Shows the PPI network extracted from STRING. b) Represents the GSVA pathway enrichment of the 13 differentially expressed genes. c) Shows a dotplot of the prognostic outcomes of each of the 13 differentially expressed genes linked to ENHO.

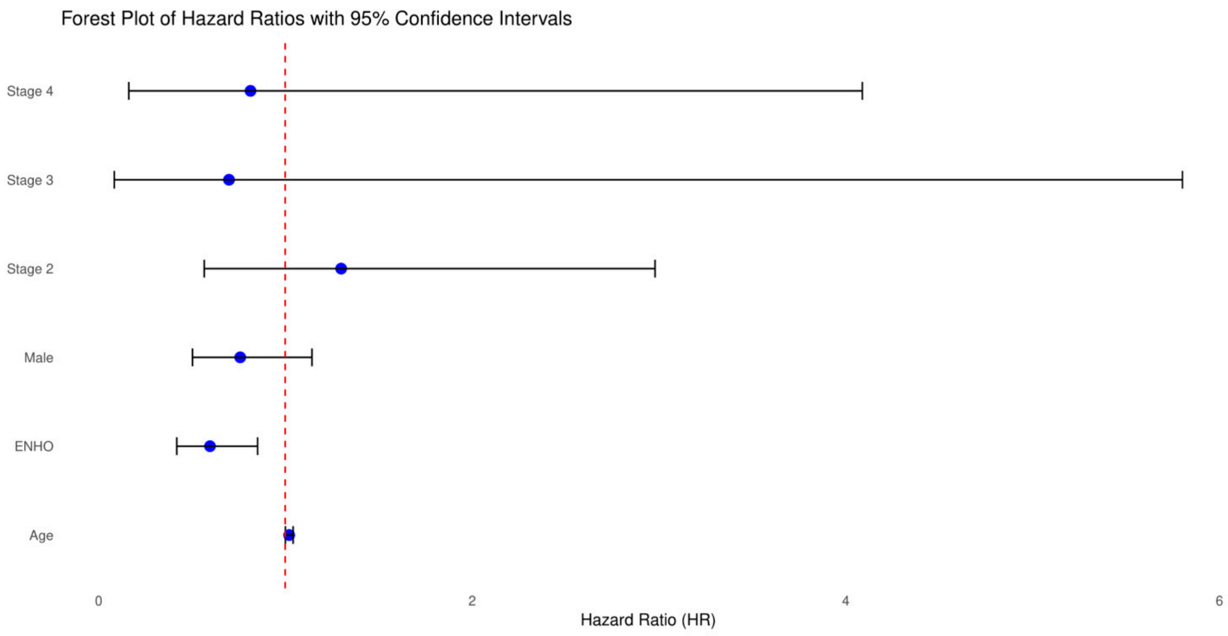

Variable	HR_CI
ENHO	0.597 (0.419–0.852)
Age	1.021 (1.001–1.042)
Stage 2	1.299 (0.566–2.98)
Stage 3	0.699 (0.084–5.803)
Stage 4	0.814 (0.162–4.089)
Male	0.759 (0.503–1.143)

Figure S4 shows the multivariate forest plot for *ENHO* expression while accounting for Stage, Age, and Gender.
