# Supplementary material for: The Role of ENHO in Pancreatic Adenocarcinoma: A Bioinformatics Approach
Source: Cancers (Basel). 2025 Jun 25;17(13):2139. doi: 10.3390/cancers17132139 (PMC12248484; doi:10.3390/cancers17132139)
Supplement: Supplementary file 1 [file cancers-17-02139-s001.zip › Tables S2 and S3.pdf]

Categorical Variables

Variable	Category	n (%)	n (%)		p-value	n (%)		p-value
		Overall	<Med	>=Med		>=Q3	<=Q1	
**Sex**	Female	82 (44.8)	35 (19.1)	47 (25.7)	0.1828	20 (20.2)	21 (21.2)	0.5784
	Male	101 (55.2)	53 (29.0)	48 (26.2)		25 (25.3)	33 (33.3)	
**Race**	Asian	12 (6.6)	5 (2.7)	7 (3.8)	0.3097	3 (3.0)	3 (3.0)	0.5385
	Black or African American	6 (3.3)	1 (0.5)	5 (2.7)		2 (2.0)	0 (0.0)	
	NR	4 (2.2)	3 (1.6)	1 (0.5)		1 (1.0)	2 (2.0)	
	White	161 (88)	79 (43.2)	82 (44.8)		39 (39.4)	49 (49.5)	
**Alcohol History**	No	66 (36.1)	31 (16.9)	35 (19.1)	0.969	17 (17.2)	21 (21.2)	0.974
	NR	12 (6.6)	6 (3.3)	6 (3.3)		3 (3.0)	3 (3.0)	
	Yes	105 (57.4)	51 (27.9)	54 (29.5)		25 (25.3)	30 (30.3)	
**Disease Type**	Adenomas and Adenocarcinomas	32 (17.5)	16 (8.7)	16 (8.7)	0.9021	12 (12.1)	10 (10.1)	N/A
	Cystic, Mucinous and Serous Neoplasms	5 (2.7)	2 (1.1)	3 (1.6)		2 (2.0)	0 (0.0)	
	Ductal and Lobular Neoplasms	145 (79.2)	69 (37.7)	76 (41.5)		31 (31.3)	44 (44.4)	
	Epithelial Neoplasms, NOS	1 (0.5)	1 (0.5)	0 (0.0)		0 (0.0)	0 (0.0)	
**AJCC Pathologic Stage**	NR	3 (1.6)	1 (0.5)	2 (1.1)	0.6573	1 (1.0)	1 (1.0)	0.3347
	Stage I	1 (0.5)	0 (0.0)	1 (0.5)		1 (1.0)	0 (0.0)	
	Stage IA	5 (2.7)	2 (1.1)	3 (1.6)		2 (2.0)	1 (1.0)	
	Stage IB	15 (8.2)	4 (2.2)	11 (6.0)		8 (8.1)	3 (3.0)	
	Stage IIA	29 (15.8)	16 (8.7)	13 (7.1)		4 (4.0)	9 (9.1)	
	Stage IIB	122 (66.7)	62 (33.9)	60 (32.8)		28 (28.3)	37 (37.4)	
	Stage III	3 (1.6)	1 (0.5)	2 (1.1)		1 (1.0)	1 (1.0)	

	Stage IV	5 (2.7)	2 (1.1)	3 (1.6)		0 (0.0)	2 (2.0)	
**Tissue/Organ of Origin**	Body of pancreas	17 (9.3)	8 (4.4)	9 (4.9)	0.3267	6 (6.1)	5 (5.1)	0.6064
	Head of pancreas	131 (71.6)	61 (33.3)	70 (38.3)		31 (31.3)	35 (35.4)	
	Overlapping lesion of pancreas	2 (1.1)	0 (0.0)	2 (1.1)		1 (1.0)	0 (0.0)	
	Pancreas, NOS	17 (9.3)	8 (4.4)	9 (4.9)		4 (4.0)	7 (7.1)	
	Tail of pancreas	16 (8.7)	11 (6.0)	5 (2.7)		3 (3.0)	7 (7.1)	
**Primary Diagnosis**	Adenocarcinoma with mixed subtypes	1 (0.5)	1 (0.5)	0 (0.0)	**0.012***	0 (0.0)	0 (0.0)	N/A
	Adenocarcinoma, NOS	23 (12.6)	15 (8.2)	8 (4.4)		4 (4.0)	10 (10.1)	
	Carcinoma, undifferentiated, NOS	1 (0.5)	1 (0.5)	0 (0.0)		0 (0.0)	0 (0.0)	
	Infiltrating duct carcinoma, NOS	145 (79.2)	69 (37.7)	76 (41.5)		31 (31.3)	44 (44.4)	
	Mucinous adenocarcinoma	5 (2.7)	2 (1.1)	3 (1.6)		2 (2.0)	0 (0.0)	
	Neuroendocrine carcinoma, NOS	8 (4.4)	0 (0.0)	8 (4.4)		8 (8.1)	0 (0.0)	
**Prior Malignancy**	No	164 (89.6)	76 (41.5)	88 (48.1)	0.1848	41 (41.4)	46 (46.5)	0.3696
	Yes	19 (10.4)	12 (6.6)	7 (3.8)		4 (4.0)	8 (8.1)	
**Prior Treatment**	No	182 (99.5)	88 (48.1)	94 (51.4)	1.000	45 (45.5)	54 (54.5)	
	Yes	1 (0.5)	0 (0.0)	1 (0.5)		0 (0.0)	0 (0.0)	
**AJCC (T)**	NR	1 (0.5)	0 (0.0)	1 (0.5)	0.1129	0 (0.0)	0 (0.0)	N/A
	T1	7 (3.8)	2 (1.1)	5 (2.7)		4 (4.0)	1 (1.0)	
	T2	24 (13.1)	7 (3.8)	17 (9.3)		11 (11.1)	3 (3.0)	
	T3	147 (80.3)	78 (42.6)	69 (37.7)		28 (28.3)	49 (49.5)	
	T4	3 (1.6)	1 (0.5)	2 (1.1)		1 (1.0)	1 (1.0)	
	TX	1 (0.5)	0 (0.0)	1 (0.5)		1 (1.0)	0 (0.0)	
**AJCC (N)**	NR	1 (0.5)	0 (0.0)	1 (0.5)	0.8861	1 (1.0)	0 (0.0)	0.7522
	N0	50 (27.3)	22 (12.0)	28 (15.3)		14 (14.1)	14 (14.1)	
	N1	123 (67.2)	62 (33.9)	61 (33.3)		27 (27.3)	38 (38.4)	
	N1b	4 (2.2)	2 (1.1)	2 (1.1)		1 (1.0)	1 (1.0)	
	NX	5 (2.7)	2 (1.1)	3 (1.6)		2 (2.0)	1 (1.0)	

**AJCC (M)**	M0	81 (44.3)	37 (20.2)	44 (24.0)	0.7822	17 (17.2)	23 (23.2)	0.4336
	M1	5 (2.7)	2 (1.1)	3 (1.6)		0 (0.0)	2 (2.0)	
	MX	97 (53)	49 (26.8)	48 (26.2)		28 (28.3)	29 (29.3)	
**Site of Resection/Biopsy**	Body of pancreas	17 (9.3)	8 (4.4)	9 (4.9)	0.3167	6 (6.1)	5 (5.1)	0.6184
	Head of pancreas	131 (71.6)	61 (33.3)	70 (38.3)		31 (31.3)	35 (35.4)	
	Overlapping lesion of pancreas	2 (1.1)	0 (0.0)	2 (1.1)		1 (1.0)	0 (0.0)	
	Pancreas, NOS	17 (9.3)	8 (4.4)	9 (4.9)		4 (4.0)	7 (7.1)	
	Tail of pancreas	16 (8.7)	11 (6.0)	5 (2.7)		3 (3.0)	7 (7.1)	
**Sample Type**	Metastatic	1 (0.5)	1 (0.5)	0 (0.0)	0.2268	0 (0.0)	1 (1.0)	0.8092
	Not Applicable	4 (2.2)	3 (1.6)	1 (0.5)		0 (0.0)	1 (1.0)	
	Primary	178 (97.3)	84 (45.9)	94 (51.4)		45 (45.5)	52 (52.5)	
**Tissue Type**	Normal	4 (2.2)	3 (1.6)	1 (0.5)	0.2687	0 (0.0)	1 (1.0)	1.000
	Tumor	179 (97.8)	85 (46.4)	94 (51.4)		45 (45.5)	53 (53.5)	

Table S2. *Distribution of clinical and pathological variables by expression quartiles and median groups, with Monte Carlo-derived p-values.* P < 0.05; NR: Not Reported. Expression metric: STAR Count.*

Note: Frequencies and column-wise percentages are shown for each category under <Med vs ≥Med and ≤Q1 vs ≥Q3 *Adropin* expression groups. P-values were calculated using Monte Carlo permutation tests with 1,000 simulations per variable. P-values are omitted (N/A) where statistical testing was not applicable due to insufficient data or zero counts.

Continuous Variables

	Mean±SD	Mean±SD		n			Mean±SD		n		
**Variable**	**Overall**	**≥Med**	**<Med**	**≥Med**	**<Med**	***p-value***	**≥Q3**	**≤Q1**	**≥Q3**	**≤Q1**	***p-value***
**Cigarettes per Day**	1.43 ± 1.00	1.43 ± 1.10	1.42 ± 0.92	28	31	0.8551	1.51 ± 1.18	1.52 ± 0.90	12	20	0.8147
**Years Smoked**	23.39 ± 14.31	22.47 ± 12.91	24.38 ± 16.04	17	16	0.9712	23.43 ± 10.63	27.92 ± 17.02	7	12	0.9325
**Age at Index**	65.01 ± 11.16	62.85 ± 10.20	66.45 ± 11.60	95	88	0.0287*	61.56 ± 9.85	66.00 ± 11.81	45	54	0.0411*
**Pack Years Smoked**	26.01 ± 18.19	26.19 ± 20.01	25.85 ± 16.71	28	31	0.8551	27.55 ± 21.47	27.65 ± 16.41	12	20	0.8147
**Initial Weight (Samples)**	245.76 ± 272.29	259.25 ± 304.49	233.19 ± 239.99	67	72	0.4707	206.67 ± 254.95	263.64 ± 280.36	33	44	0.0399*
**Intermediate Dimension (Samples)**	0.76 ± 0.31	0.70 ± 0.19	0.71 ± 0.32	28	16	0.902	0.67 ± 0.20	0.69 ± 0.27	12	10	0.8154
**Shortest Dimension (Samples)**	0.33 ± 0.12	0.36 ± 0.13	0.30 ± 0.11	28	16	0.1282	0.38 ± 0.15	0.29 ± 0.09	12	10	0.1954
**Longest Dimension (Samples)**	1.25 ± 0.40	1.20 ± 0.32	1.18 ± 0.49	28	16	0.5689	1.04 ± 0.14	1.22 ± 0.51	12	10	0.4585
**Overall Survival Time (Days)**	565.09 ± 463.60	667.53 ± 513.42	454.45 ± 396.51	95	87	0.0006*	785.73 ± 604.75	472.94 ± 392.20	45	53	0.0011*

Table S3. *Comparison of Continuous Variables Across Median and Quartile Groups Using Wilcoxon Rank-Sum Test. . Expression metric: STAR Count.*

This table presents the comparison of continuous clinical and sample-related variables stratified by the median (Med) and quartiles (Q1 and Q3). Mean ± standard deviation (SD) values are shown for each group. P-values are derived from the Wilcoxon rank-sum test, assessing differences between high vs. low median groups and between upper (≥Q3) and lower (≤Q1) quartile Adropin expression groups. Asterisks (*) indicate statistically significant differences ($p < 0.05$). Variation in sample sizes (“n”) across comparisons reflects missing values (“NA”) excluded from analyses. Quantile cutoffs were based on all samples with reported expression data (n = 183).
